# Prevalence of oral pain and barriers to use of emergency oral care facilities among adult Tanzanians

**DOI:** 10.1186/1472-6831-8-28

**Published:** 2008-09-29

**Authors:** Emil Namakuka Kikwilu, Joyce Rose Masalu, Febronia Kokulengya Kahabuka, Ahadieli Raphael Senkoro

**Affiliations:** 1 Department of Preventive and Community Dentistry, School of Dentistry, Muhimbili University of Health and Allied Sciences, P.O. Box 65014, Dar es Salaam, United Republic of Tanzania; 2 Central Oral Health Unit, Ministry of Health, United Republic of Tanzania

## Abstract

**Background:**

Oral pain has been the major cause of the attendances in the dental clinics in Tanzania. Some patients postpone seeing the dentist for as long as two to five days. This study determines the prevalence of oral pain and barriers to use of emergency oral care in Tanzania.

**Methods:**

Questionnaire data were collected from 1,759 adult respondents aged 18 years and above. The study area covered six urban and eight rural study clusters, which had been selected using the WHO Pathfinder methodology. Chi-square tests and logistic regression analyses were performed to identify associations.

**Results:**

Forty two percent of the respondents had utilized the oral health care facilities sometimes in their lifetime. About 59% of the respondents revealed that they had suffered from oral pain and/or discomfort within the twelve months that preceded the study, but only 26.5% of these had sought treatment from oral health care facilities. The reasons for not seeking emergency care were: lack of money to pay for treatment (27.9%); self medication (17.6%); respondents thinking that pain would disappear with time (15.7%); and lack of money to pay for transport to the dental clinic (15.0%). Older adults were more likely to report that they had experienced oral pain during the last 12 months than the younger adults (OR = 1.57, CI 1.07–1.57, *P *< 0.001). Respondents from rural areas were more likely report *dental clinics far from home (OR = 5.31, CI = 2.09–13.54, P < 0.001); self medication at home (OR = 3.65, CI = 2.25–5.94, P < 0.001); *and *being treated by traditional healer (OR = 5.31, CI = 2.25–12.49, P < 0.001) *as reasons for not seeking emergency care from the oral health care facilities than their counterparts from urban areas.

**Conclusion:**

Oral pain and discomfort were prevalent among adult Tanzanians. Only a quarter of those who experienced oral pain or discomfort sought emergency oral care from oral health care facilities. Self medication was used as an alternative to using oral care facilities mainly by rural residents. Establishing oral care facilities in rural areas is recommended.

## Background

In Tanzania, most of the oral health care is rendered at government owned dental clinics, which are established within the hospitals in the regional and district capitals. Patients from rural areas have to travel to towns to seek dental services. Before 1994, the health care services, oral health included, were fully funded by the government. Thereafter, the policy on cost sharing in health was introduced, by which patients aged six years and above are required to pay for their health care. Along with this policy, patients age 60 years old and above and any younger patient who is confirmed to be poor that he/she is unable to pay for health care also receives service free of charge. Since documentation for age and personal/family income is not well developed in Tanzania, the exception criterion based on age or inability to pay presents a challenge to hospital administrators. A study which compared the attendance data one year before and after the introduction of cost sharing in health revealed an overall drop of 33% in attendances, indicating a negative effect on the utilization of oral health services in Tanzania [1].

Oral pain has been the major cause of attendances in all dental clinics in Tanzania [2]. A review of patient records at the School of Dentistry, Dar es Salaam indicated that the majority of patients who had attended the dental outpatient clinics during the month of March 2007 complaining of pain had delayed for two to three days before they reported for treatment. The negative impacts of orofacial pain on quality of life have been documented as substantial in several studies [3-6]. The major negative impacts of orofacial pain include physical and psychosocial disabilities to the persons afflicted [3-6], and to their associates [6].

Reducing the episodes and the duration of oral pain is one of the objectives of oral health care in Tanzania. To achieve this objective, oral health care planners and providers will, among other undertakings, require a better understanding of the barriers to prompt seeking of emergency oral care due to oral pain. Once the barriers to prompt seeking of emergency oral care have been identified and addressed, people would likely seek emergency oral care without delays and therefore reduce the duration of suffering from oral pain. This would in turn contribute to the improvement in the quality of life and the well being of Tanzanians, which has been identified as one of the broad outcome measures for reduced poverty and improved economic growth for Tanzania [7].

Literature on oral pain and the utilization of oral health care in Tanzania indicates that all the previous studies reported high proportions of patients who sought care due to oral pain. A report by van Palenstein and Nathoo shows that in 1989, 77% to 97% of all the dental attendances in Tanzania were due to oral pain [8]. In 1993, Mosha *et al*. [9] reported that on average, 82% of all the adults who had attended the oral health care facilities in Tanzania had sought emergency oral care at least once during their lifetime. In an interview of families who lived in a catchment area of a rural health centre which had rendered emergency oral care for two years, 40% of all families interviewed had one or more of their members who had used the service within the two years of its establishment [9,10].

None of the publications quoted in the preceding paragraph reported on the prevalence of oral pain in the community or on the barriers to the use of the emergency oral care facilities. In their report, van Palenstein *et al*. [8] pointed out that the hospital data might be showing only a tip of the iceberg of all the people who suffered from oral pain in communities. The aim of this study was to determine the prevalence of oral pain and/or discomfort and the barriers to use of the emergency oral care facilities due to oral pain or discomfort among adult Tanzanians.

## Methods

### Selection of the study sites and Sampling procedure

Determining the sample size and selecting the study sites were done according to the national pathfinder survey methodology which is described in the WHO Oral Health Surveys – Basic Methods [11]. In this WHO publication, inclusion of study sites from each of the country's administrative zones is recommended. In mainland Tanzania there were six administrative zones, of which two were earmarked as urban and four as rural study sites. Six clusters from the urban study sites and eight clusters from the rural study sites were purposively selected for this study. The clusters were wards and villages for urban and rural sites respectively. From each of the clusters selected, 150 adults aged 18 years and above were to be interviewed. Therefore, a total of 2,100 subjects (900 urban, 1200 rural) were targeted. To facilitate the stratification of the respondents by sex and age, each interviewer was instructed to interview equal numbers of males and females for each of the following five age-groups: 18–25, 26–35, 36–45, 46–55, and 56+. By the end of the study period, only 1,759 out of the targeted 2,100 respondents were interviewed, giving a response rate of 84%.

### Procedure used to select study participants

This study was a house-to-house survey. Cities and villages in Tanzania are divided into small administrative units of 10–20 households called streets. Interviewers reported to the city, or village authorities who assigned one street leader to lead the interviewers from house to house under his/her street until all the adults in a given street, who were present at the time of the study, were interviewed. After one street had been done, the respective street leader passed over the responsibility of leading the interviewers to the next street leader. This process continued until the interviewers had interviewed the required number of adults of each age-group and sex category.

### Ethical clearance and procedure for obtaining informed consent from respondents

The ethical clearance for conducting this study was obtained from the Ministry of Health of the United Republic of Tanzania. The street leaders who led the interviewers introduced them to the family members of each household, after which the interviewers explained the aim of the visit. After the household members had understood the aim of the study, all members aged 18 years and above were requested to participate in the study by responding to the questions posed by interviewers. Members were informed that participation in the study was absolutely voluntary.

### Development of a questionnaire

The data reported in the current study was part of the national pathfinder survey that aimed at exploring the use of oral health services, satisfaction with the last dental visit, risk oral health behaviours, perception on oral health care and oral health care providers and oral health related quality of life. The authors could not get a validated questionnaire that could be used to study these aspects of oral health in Tanzania, therefore a questionnaire had to be developed. The questionnaire was constructed from a list of oral health indicators summarized in the report of the *Consensus Workshop for Selecting Essential Oral Health Indicators in Europe *held at the University of Granada, Spain [12]. It was pre-tested among 20 adults in each of the six administrative zones for meaning and clarity. A meeting of the interviewers was convened to discuss the results of the pilot. Words and sentences that were not clear or seemed to distort the meaning of questions were changed. The revised questionnaire was administered to a randomly selected group of 35 adults to check for clarity. The same questionnaire was administered to the same group of adults after one week for reliability testing. This report deals with five questions on the use of the emergency oral care facilities.

### Data analysis

#### Validity and Reliability

The test retest correlation coefficients for individual questions ranged from 0.80–1.0. These correlation coefficients were evidence of the high reliability of the questionnaire.

#### Coding of variables

The data were entered and analysed using SPSS version 11.5. Independent variables were dummy coded as follows: *Residence *(0 = urban, 1 = rural); *Sex *(0 = male, 1 = female); *age *was dichotomized into young adults (18–40 yrs) and older adults (41+yrs), then coded as (0 = young adults, 1 = older adults); *education *was dichotomized into less educated (primary level or below) and educated (secondary level and higher) then coded as (0 = educated, 1 = less educated). A category coded '0' was taken as a reference category in the logistic regression analyses. Dependent variables *pain experience; lack of money for treatment; lack of money for transport; dental clinic far from home; self medication; thought pain would disappear; was treated by traditional healer; and no substantial reason *were coded (0 = No, 1 = Yes). Categories coded 1 were taken as the outcomes of interest in the logistic regression analyses.

### Statistical analysis

The frequency distributions of respondents by the responses to the five key questions on oral pain, seeking emergency oral care and reasons for not seeking emergency oral care were generated. A Chi-square test was performed to identify associations between independent and dependent variables. Dependent variables that showed statistically significant Chi-square values at p < 0.05 were each entered in a multivariate logistic regression model using the four independent variables as predictor variables.

## Results

A total of 1,759 adults aged 18 – 92 years old (mean = 40 yrs; SD = 16.3), were interviewed about their experiences with oral pain and their use of dental services. The proportions of respondents by demographic characteristics are summarized in Table 1. There was a statistically significant difference between place of residence and the level of education of subjects (χ^2 ^= 142.1, *P *< 0.0001). The distributions of respondents by sex and age-groups were independent to residence.

**Table 1:** Percent distribution of respondents by residence, sex, age, and education and their associated χ^2^- and *p*-values

residence	sex		Age-groups		Education level	
	male	female	young adults (≤ 40 years)	older adults (41+ years)	Primary & lower	Secondary & above
Urban (n = 732)	50.5	49.5	60.0	40.0	70.7	29.3
Rural (n = 1027)	48.0	52.0	55.7	44.3	92.2	7.8
Total (1759)	49.1	50.5	57.5	42.5	83.3	16.7
χ^2 ^-test for independence	χ^2 ^= 2.06, *P *= 0.151		χ^2 ^= 3.3, *P *= 0.069		χ^2 ^= 142.1, *P *< 0.0001	

The responses to five questions on oral pain experience and use of dental services in the dental clinics are summarized in Tables 2 and 3. Only 42.1% of the interviewed adults had been treated in a dental clinic during their lifetime. Nevertheless, 58.8% had experienced oral pain or oral discomfort during the last 12 months before the interview, of which 26.5% had sought treatment from dental clinics. Those who reported having no substantial reasons for not seeking emergency oral care at a dental clinic were 31.3%. The common reasons that were given by the respondents for not seeking emergency care were: *financial constraints, self medication, wait and see behaviour or thinking that the problem would disappear, fear of pain during treatment, long distances from residence to dental clinic*, and *use of traditional healers*.

**Table 2:** Distribution of respondents by responses to different questions on service utilization and experiences with oral pain

Specific question and response options	Number	Percentage
Have you ever been treated in a dental clinic?		
Yes	741	42.1
No	1018	57.9
If yes, when did you last get treated in a dental clinic?		
< 6 months	102	13.8
6 – 12 months	184	24.8
> 1 – 2 years	97	13.1
> 2 but < 5 years	125	16.9
5+ years	233	31.4
Did you experience any oral pain or discomfort during the last one year?		
Yes	1034	58.8
No	725	41.2
Did you get treated in the dental clinic when you had pain or discomfort during the last one year?		
Yes	274	26.5
No	760	73.5

**Table 3:** Distribution of 760 respondents who did not seek oral emergency care at the dental clinics by reasons for not doing so

Reasons for not seeking oral emergency care at dental clinics	Number	Percent
No substantial reason	238	31.3
I could not afford fee for treatment	212	27.9
I had self medication	134	17.6
I thought the problem will disappear	119	15.7
I could not get money for transport to dental clinic	114	15.0
I was treated by a traditional healer	59	7.6
Dental clinic too far from my home	49	6.4
I fear dental treatment	42	5.5
Dental clinic not reachable	4	0.5

Table 4 shows the multivariate logistic regression analyses with odds ratios (95% C.I.) for oral pain experience during the last 12 months prior to the study and for reasons that prevented patients from seeking emergency care by independent variables residence, sex, age and education. Women (OR = 1.30, CI = 1.07–1.57, p < 0.01), older adults (OR = 1.57, CI = 1.29–1.91, p < 0.001), and less educated respondents (OR = 1.34, CI = 1.03–1.75, p < 0.05) were more likely to report that they had experienced oral pain or discomfort during the last 12 months preceding the interview than men, young adults and the educated respondents respectively.

**Table 4:** Multiple Logistic regression Odds ratios (95% CI) for oral pain experience during last 12 months and reasons for not seeking oral emergency care by independent variables residence, sex, age and education

	Independent variables^§^			
Dependent variables^#^	Residence (*urban*/rural)	Sex (*male*/female)	Age-groups; (*young*/older)	Education (*educated*/less educated)

Pain during the last 12 months	1.19 (0.97–1.45)	1.30 (1.07–1.57)**	1.57 (1.29–1.91)***	1.34 (1.03–1.75)*
*Reasons for not using oral care facilities*				
- Lack of money for treatment	1.10 (0.81–1.51)	1.37 (1.03–1.83)*	1.36 (1.01–1.81)*	1.60 (0.98–2.63)
- Lack money for transport	2.12 (1.34–3.36)**	1.07 (0.73–1.55)	1.51 (1.04–2.20)*	1.89(0.89–4.01)
- Dental clinic far from home	5.31 (2.09–13.54)***	0.72 (0.42–1.25)	2.80 (1.59–4.93)***	2.91 (0.69–12.33)
- Self medication at home	3.65 (2.25–5.94)***	1.37 (0.96–1.95)	1.36 (0.95–1.93)	1.51 (0.76–2.99)
- Thought pain will disappear	0.84 (0.58–1.22)	0.76 (0.53–1.09)	0.85 (0.59–1.24)	1.45 (0.81–2.58)
- Treated by traditional healer	5.31 (2.25–12.49)***	0.69 (0.41–1.15)	1.07 (0.64–1.79)	3.43 (0.82–14.41)
- No substantial reason	0.58 (0.45–0.75)***	0.68 (0.54–0.85)**	0.61 (0.48–0.78)***	0.64 (0.45–0.92)*

§ = independent variables coding (italicised category = 0; non-italicised category = 1)

# = dependent variables coding (*no = 0; yes = 1); with "yes" as an outcome of interest*

*P < 0.05; **P < 0.01; ***P < 0.001

Respondents from rural areas were more likely to report *lack of money for transportation to dental clinics *as a reason for not seeking emergency care from the oral health care facilities than their counterparts from urban areas (OR = 2.12, CI = 1.34–3.36, P < 0.01). They were also more likely report *dental clinics far from home (OR = 5.31, CI = 2.09–13.54, P < 0.001); self medication at home (OR = 3.65, CI = 2.25–5.94, P < 0.001); *and *being treated by traditional healer (OR = 5.31, CI = 2.25–12.49, P < 0.001) *as reasons for not seeking emergency care from the oral health care facilities than their counterparts from urban areas. Moreover respondents from rural areas were less likely to report that they had *no substantial reasons *for not seeking emergency care from the oral health care facilities than urban residents (OR = 0.58, CI = 0.45–0.75, *P *< 0.001).

Women were more likely to report that they *had no money for treatment *than men (OR = 1.37, CI = 1.03–1.83, *P *< 0.05). Also women were less likely to report no substantial reason for not seeking emergency care from oral care facilities than men (OR = 0.68, CI = 0.54–0.85, P < 0.01). Older adults were more likely to report *dental clinic far from home *as a reason for not seeking emergency oral care than young adults (*OR = 2.80, CI = 1.59–4.93, P < 0.001*). They were also more likely to report *lack of money for treatment (OR = 1.36, CI = 1.01–1.81, P < 0.05) *and/or *transport to clinic (OR = 1.51, CI = 1.04–2.20, P < 0.05) *as reasons for not seeking emergency oral care than young adults. Also older adults were less likely to report that they *had no substantial reason *for not seeking emergency care than young adults (*OR = 0.61, CI = 0.48–0.78*, p < 0.001).

## Discussion

The interpretation of the data reported in this study should be done with the following methodological limitations in mind: First, probability sampling was not used to obtain study clusters. Secondly, only adults who were present at their homes during the study hours were interviewed. Although random sampling was used to obtain regions for inclusion into the study and the study hours adjusted to suit times when most of adults were expected to be present at their homes, one cannot say with certainty that the data is representative of adults in Tanzania. Nevertheless, the strategy of moving from one household to another and interviewing adults at their homes minimized the selection bias that would arise from calling respondents to one location in a street. Calling respondents to one location in a street would have included, in the study, only adults who were enthusiastic and ready to participate in joint street activities, while leaving out adults who were less receptive. This also controlled the selection bias that could have arisen from street leaders selecting the households of their interest. In addition, the fact that each administrative zone was represented took care of the possible variations between zones. Furthermore, the predetermined number of respondents in each sex and age-group ensured that both sexes and age-groups were fairly represented in the study. Given all these control measures, the authors were satisfied that the findings reported in the present study were reliable enough to be used for planning purposes.

The prevalence of oral pain reported in the current study (58.8%) was higher than those reported in Brazil, Nigeria, and Burkina Faso which were 39.9%, 34%, and 27.7% respectively [4,13,14]. The proportion of respondents (26.5%) who used the emergency oral health care facilities following an episode of oral pain in the present study is similar to that reported among adults in Burkina Faso (27.7%) [14]. In the present study, lack of money was the most frequently reported barrier to seeking emergency oral care. This finding is similar to that reported among rural villagers in Tanzania, adult population in Burkina Faso and among University students in Kenya [10,14,15]. Similar findings were also reported among underserved communities in USA [16-18]. These findings indicate that cost may be a major barrier for seeking oral care in many communities whose earnings just meet the bare minimum of life.

The finding that 58.8% of all respondents had experienced some episodes of oral pain or discomfort during the 12 months preceding the interview, indicate that oral health problems are common among adults in Tanzania. Since only 26.5% of those who experienced some episodes of oral pain or discomfort sought emergency oral care in oral health care facilities, it can be extrapolated that if all adult Tanzanians who experience oral pain or discomfort would seek emergency oral care in oral health care facilities, the current workload in these facilities would triple, and would necessitate an increase in the facilities and human resources to meet such demand.

About a third of respondents who had not sought emergency oral care despite having experienced oral pain or discomfort during the last 12 months leading to the study, gave no substantial reasons for not seeking such service. These might have had only mild discomfort that did not require emergency oral care. It might also mean that pain disappeared before they were able to seek treatment. Similar response could also have been elicited from adults who tolerated oral pain due to other competing needs that involved time and finances. It is on this account that it is worth noting that the questionnaire did not adequately capture the reasons why some respondents did not have 'substantial reasons' for not seeking emergency oral care. It is hereby recommended that future studies on reasons for not seeking emergency oral care should be undertaken to address this shortfall.

About a third of the respondents could not seek emergency oral care because of financial reasons. The Ministry of Health should make people aware of the provision for exemption for those who cannot pay for these services. This will allow more people who cannot afford the treatment fees to access emergency oral care. It is hoped that the current government's efforts aimed at reducing poverty and increasing the economic growth for all Tanzanians are likely to reduce the identified barriers for seeking emergency oral care. This is because higher economic status has been shown to correspond with high utilization of oral health services [14]. There is also a need to raise awareness among Tanzanians on the importance of prompt seeking of oral care to prevent adverse sequel of advanced oral diseases. This is particularly important because, as already mentioned, 1/3 of those who had had pain or discomfort reported to have no substantial reasons for not seeking emergency oral care. In addition, a study among the people in one region in Tanzania did reveal that not all people in Tanzania who report to have not utilized health services due to financial reasons are actually unable to pay for the service. Rather, they use the pretext of being poor as a convenient way of explaining away their negligence [19].

The findings that women were more likely to report that they had experienced oral pain than men is likely to be due to cultural traditions. In Tanzania, men tend to shy away from reporting pain because it is generally taken as 'normal' for a man to tolerate pain. This observation conforms to what has been reported in other countries [20-22]. This may also explain why more men than women did not seek emergency oral care on the assumption that pain would disappear spontaneously.

People who live in rural areas were disadvantaged in relation to accessing the emergency oral care from oral care facilities compared to those who live in urban areas. In the present study, respondents from rural areas were more likely than respondents from urban areas to give reasons such as distance from their home, lack of money for transportation to dental clinics; being treated by traditional healers; and using medicines at home for not seeking emergency oral care from oral care facilities. Since oral pain and discomfort were equally prevalent among rural and urban residents, there is a need for establishing oral health care services in rural health centres and dispensaries.

## Conclusion

From the present study, we conclude that: a) the prevalence of oral pain or discomfort was equally high among rural and urban adult Tanzanians; b) only a quarter of those who had experienced episodes of oral pain or discomfort sought emergency oral care from the oral care facilities; and c) proportionately more rural residents reported more barriers to the use of oral care facilities than did urban residents. The Ministry of Health should consider establishing oral health care services in rural areas to improve accessibility of oral care facilities by rural residents. Because a third of the respondents did not give reasons for not seeking emergency oral care despite having experienced oral pain, further studies are needed to better understand the oral health care seeking behaviour among adult Tanzanians.

## Competing interests

The authors declare that they have no competing interests.

## Authors' contributions

EK participated in conception and design of the study, data analysis and interpretation, and drafting the manuscript. JM participated in the conception and design of the study, acquisition of data and critical revision of manuscript. FK critically reviewed the proposal and composition of the questionnaire, acquisition of data and critical review of manuscript. AS formulated the research question, conception of the study, acquisition of data and critically reviewing the manuscript.

## Pre-publication history

The pre-publication history for this paper can be accessed here:


